# The effect of deforestation on COVID-19 transmission to Indigenous peoples in Brazil: A panel fixed-effects analysis before and after vaccination

**DOI:** 10.1371/journal.pgph.0004527

**Published:** 2025-04-29

**Authors:** Humberto Laudares, Carolina Batista, Pedro Henrique Gagliardi, Rudi Rocha, Nicolas Ray

**Affiliations:** 1 Faculty of Medicine, GeoHealth Group, Institute of Global Health, University of Geneva, Geneva, Switzerland; 2 Drugs for Neglected Diseases Initiative (DNDi) Latin America, Rio de Janeiro, Brazil; 3 Columbia University, New York, United States of America; 4 São Paulo School of Business Administration, Fundação Getulio Vargas, São Paulo, Brazil; 5 Institute for Environmental Sciences, University of Geneva, Geneva, Switzerland; University of the Southern Caribbean, TRINIDAD AND TOBAGO

## Abstract

Brazil had the second-largest death toll during the COVID-19 pandemic, with indigenous peoples disproportionately affected among ethnic groups. Parallel to the pandemic, Brazil has recorded the highest rate of deforestation globally, with encroachments into Indigenous territories putting climate stabilization and biodiversity at risk. However, the effects of deforestation on COVID-19 transmission to Brazil’s Indigenous peoples are unknown. This study shows that during the pre-vaccination period, deforestation partially explains COVID-19 transmission among Indigenous populations. Our main results for the pre-vaccination period indicate that a daily increase in deforestation per km^2^ is associated, on average, with the confirmation of 0.76 (p < 0.004, 95% CI: 0.240 - 1.276) new daily cases of COVID-19 among Indigenous peoples 14 days after deforestation warnings. Our estimates suggest deforestation explains at least 9.6% of all COVID-19 cases among indigenous populations. The association between the two variables disappears after the vaccination program. Our findings provide empirical evidence on the interplay between environmental degradation and negative health outcomes in a vulnerable segment of society in the context of a pandemic. Furthermore, these findings highlight the importance of the One Health approach to building preparedness for future pandemic threats.

## Introduction

Brazil ranked second globally in the number of lives lost during the COVID-19 pandemic. However, the negative impact of the pandemic was unevenly distributed across the population, with Indigenous peoples experiencing one of the highest mortality rates among ethnic groups [1–4]. Policy support for vulnerable groups, including Indigenous communities, was deprioritized [2,5]. As a result, the Brazilian Supreme Court intervened in October 2020, mandating the federal government to take urgent action to contain the spread of COVID-19 in indigenous territories. When vaccination began in Brazil in mid-January 2021, Indigenous communities were prioritized due to their heightened vulnerability. Within two months, approximately 47.9% of the Indigenous population aged 19 or older had received the first dose of the vaccine.

In parallel to the rapid spread of COVID-19, Brazil has recorded the highest rate of deforestation in the world, threatening climate stabilization and biodiversity [5]. From April 2020 to September 2021, 21,958 km² of land was deforested—equivalent in size to countries such as Djibouti, Belize, or Israel. Alarmingly, one-third of this deforestation took place on Indigenous lands, accounting for 30% of total deforestation in the country, a dramatic increase from the historical average of 1.6% between 1985 and 2020 [6].

The acceleration of deforestation since 2018 has been accompanied by an upsurge in illegal economic activities such as gold mining and land grabbing, exacerbating conflicts between indigenous and non-indigenous groups. Federal policies have actively facilitated this process; during the first six months of the pandemic alone, the government enacted 28 pieces of legislation to weaken environmental laws, proposed a bill to allow mining on Indigenous lands, and reduced environmental fines by 72%, fueling rapid deforestation [7].

Deforestation, especially in rainforest regions such as the Amazon, has intensified human-wildlife interactions, heightening the risk of zoonotic spillover, where a virus is transmitted from animals to humans [8,9]. The destruction of forest habitats displaces wildlife species, forcing them into closer proximity to human populations and increasing the likelihood of pathogen transmission. This phenomenon has been observed globally, with studies highlighting that deforestation contributes to the proliferation of disease vectors, ecosystem instability, and the emergence of infectious diseases [10,11]. The emergence of the coronavirus (SARS-CoV-2) is an example of zoonotic spillover. The case of Indigenous communities in Brazil exemplifies this trend, as environmental degradation compounds their existing vulnerabilities, increasing exposure to existing infectious diseases and also zoonotic threats while simultaneously diminishing their access to traditional medicine and healthcare services [12,13]. Deforestation can affect Indigenous communities through zoonotic spillovers but also through social conflicts, exposure to non-Indigenous extractive economic activities, and land grabbing.

The One Health approach provides a critical framework for understanding and mitigating these interrelated threats by integrating human, animal, and environmental health perspectives [8]. Most of the academic literature on One Health is still focused on human-animal health [14]. While One Health might offer a conceptual opening to integrating Indigenous knowledge of health, more research is needed on this topic [11,15,16]. Overall, One Health offers a framework to mitigate the compounded effects of deforestation and pandemics on vulnerable populations.

This study empirically assesses whether and to what extent deforestation has been associated with increased COVID-19 rates among Brazil’s indigenous peoples before and after the vaccination program rollout. By applying a One Health lens, this research underscores the necessity of interdisciplinary approaches to pandemic preparedness, environmental governance, and indigenous health protection in the face of accelerating ecological and epidemiological crises.

## Methods

### Data sources

We used several publicly available datasets for our observational analysis (S1–S3 Tables for summary statistics). The municipal-level panel data includes 1,802,052 observations from all 5,570 Brazilian municipalities over 579 days. The Indigenous health subdistrict (*pólo base*) panel data covers the same period, comprising 362 subdistricts within 34 Special Sanitary Districts (ISSD), totaling 209,598 observations. Our dataset spans 01 April 2020—the date of the first reported COVID-19 case among Indigenous peoples—until 30 September 2021, coinciding with the start of the third vaccine dose rollout.

The primary variables are: i) COVID-19 confirmed cases among Indigenous peoples and ii) deforestation alerts. First, we collected COVID-19 data from the Special Department of Indigenous Health (SDIH) of the Ministry of Health. The dataset was organized by the Indigenous Special Sanitary Districts (ISSD), decentralized administrative healthcare units that serve indigenous populations. The data was also broken down by Indigenous health subdistricts (*Pólo base*). There are 34 ISSDs within the boundaries of 220 municipalities. Since ISSD or Indigenous health subdistrict data is aggregated across multiple municipalities, we estimated COVID-19 cases at the municipality level using a proportional allocation approach. We multiplied the ratio of the municipality population over the total respective ISSD population by the total number of COVID-19 cases per ISSD. Second, we collected data on deforestation hotspots at the municipality level from the National Institute for Space Research, including georeferenced deforestation alerts in the Amazon and the Cerrado biomes.

We performed our primary analysis using data from both Indigenous health subdistricts and municipal levels. The panel data format allowed us to use the temporal and spatial variation in between deforestation and COVID-19 transmission to access their relationship.

We also used the data in a cross-sectional format to explore potential mediator factors. We analyzed economic, social, geographic, and health-related factors that could act as potential mediators. To minimize possible bias due to omitted variables in our cross-section analysis, we included controls in the regression models: wildfire, cattle ranching, rainfall, and latitude.

We also performed a robustness check to verify whether the link between deforestation and COVID-19 was specific to Indigenous peoples. We analyzed the Severe Acute Respiratory Syndrome (SRAG) dataset from the Ministry of Health (2019–2020). This individual-level dataset includes self-reported racial identity (White, Mixed/*Pardo*, Asian, Black) and COVID-19 status. This allowed us to assess whether deforestation also influenced infection rates in non-Indigenous populations.

### Variables

The main variables used in the article are summarized in the table below. S1 File in the Annex provides more details of those variables.

Table 1 summarizes all the variables used in the panel data and cross-section analyses.

**Table 1:** Summary of the variables used in the analyses.

Variables	Description	Frequency	Source
COVID-19 cases for Indigenous people	COVID-19 cases for indigenous peoples reported by Indigenous health subdistricts and estimated to municipalities	Daily	Brazilian Unified Health System (SUS), Indigenous Health Department
COVID-19 cases for other racial groups	COVID-19 cases registered at municipal level	Daily	Brazilian Unified Health System (SUS), SRAG dataset
Deforestation (in km^2^)	Number of warning areas for deforestation (per km^2^) at the municipal level	Daily	National Institute for Space Research (INPE)
Vaccination (1st dose)	Daily number of indigenous people vaccinated against COVID-19 (1st doses)	Daily	National Vaccination Campaign against COVID-19, Brazilian Unified Health System (SUS)
Vaccination (2nd dose)	Daily number of indigenous people vaccinated against COVID-19 (2nd doses)	Daily	National Vaccination Campaign against COVID-19, Brazilian Unified Health System (SUS)
Rainfall	Average precipitation per municipality	Daily	Xavier, A. C., Scanlon, B. R., King, C. W., & Alves, A. I. (2022)
Wind speed	Average wind speed per municipality	Daily	Xavier, A. C., Scanlon, B. R., King, C. W., & Alves, A. I. (2022)
Existence of conflicts between Indigenous and non-Indgenous	Number of registered occurances of conflicts between Indigenous and non-Indigenous people	Total period	Comissão Pastoral da Terra
Illegal mining (2019–2020)	Expanded illegal mining areas in 2019 and 2020 in m2	Total period	Mapbiomas
Growth in illegal mining during 2019–2020	Growth in illegal mining area from 2019-2020 relatively to 2017–2018	Total period	Mapbiomas
Wildfires	Fire Radiative Power (FRP), which is the measurement of the radiant energy released per time unit by burning vegetation per 1,000 km^2^	Annual	Burning Program of the National Institute for Space Research (INPE)
Cattle ranching	Total number of bovine cattle by municipality per 1,000 km^2^ in 2019. Natural logarithmic form.	Annual	Brazilian Institute of Geography and Statistics (IBGE)
Rainfall	Average of rainfall at the municipal level in millimeters per hour (mm)	Annual	Brazilian Institute of Geography and Statistics (IBGE)
Waterways	Binary variable for municipalities in which their centroids are at least 100 km distance to the nearest waterway.	Fixed	Brazilian Institute of Geography and Statistics (IBGE)
Latitude	Municipalities or Indingeous heath subdistricts’Latitude	Fixed	Brazilian Institute of Geography and Statistics (IBGE) and Authors’ own calculation with ArcGis
Size of Indigenous health teams	The average size of Indigenous health teams from March to June 2020	Annual	Ministry of Health
Number of hospitals	Number of hospitals	Annual	Ministry of Health
GDP per capita (2017)	Gross Domestic Production (GDP) per capita (2017)	Annual	Brazilian Institute of Geography and Statistics (IBGE)
Income inequality (Gini coefficient)	Income inequality by municipality measured by the Gini Index	Annual	Brazilian Institute of Geography and Statistics (IBGE)
Human Development Index (HDI)	The geometric mean of normalized indices captures three dimensions: a long and healthy life, being knowledgeable, and having a decent standard of living. Data from 2010.	Annual	United Nations Development Programme (UNDP)
Expenditure related to COVID-19	The total amount transferred from the central government to the local government during the COVID-19 pandemic in 2020.	Annual	Ministry of Finance

### Statistical analysis

The empirical analysis estimated the association between deforestation per km^2^ and daily COVID-19-confirmed cases among Indigenous peoples at the municipality and Indigenous health subdistrict level. We estimated equations that follow the form:


$$COVID_{it}=\alpha +\beta {Deforestation}_{i(t-l)}+\lambda_{i}+\delta_{t}+\upsilon_{it},$$ (1)

where COVID_*it*_ is the dependent variable capturing the number of COVID-19 cases among Indigenous peoples in the municipality *i* in time period *t.* Deforestation _*i(t-l)*_ is the main variable of interest, representing deforestation alerts per km^2^ in the municipality, lagged by 𝑙 days. The term λ_*i*_ are municipality (or Indigenous health subdistrict) fixed effects, controlling for time-invariant confounders such as climate, geography, and infrastructure. The term δ_*t*_ refers to time fixed-effects, capturing common temporal trends in deforestation and COVID-19 dynamics. The error term is expressed by υ_*it*_, absorbing all other omitted factors.

Our primary specification uses a 14-day lag (*l* = 14), with additional estimations at 6 days (S2 Fig). The rationale for this choice is as follows. First, epidemiological evidence showed that the median symptom onset period is 11.5 days after infection, with an average incubation period of 5.1 days [17]. Second, delayed testing and diagnosis was a reality in context investigated, given geographic barriers and limited healthcare access among Indigenous populations. Finally, we can eliminate contemporaneous effects using lagged deforestation variables, which helps mitigate potential reverse causality between COVID-19 reporting and deforestation activities. We also performed a sensitivity analysis by testing different lag structures (Fig 5).

**Fig 1:**
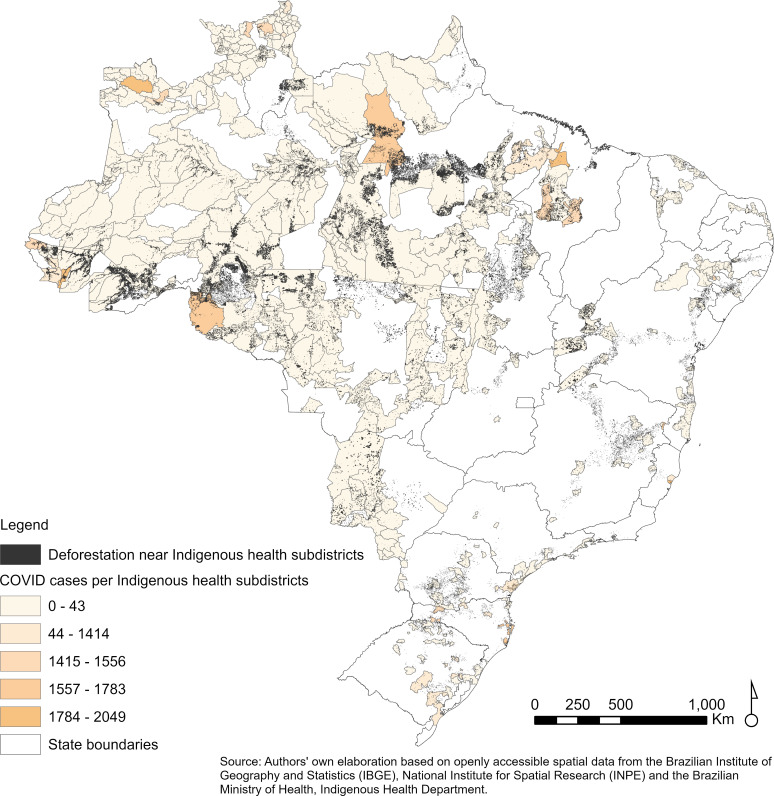
COVID-19 cases and deforestation in Indigenous health subdistricts. The map shows the number of confirmed COVID-19 cases among Indigenous peoples in Brazil and deforestation hotspots from April 1, 2020, to September 30, 2021.

**Fig 2:**
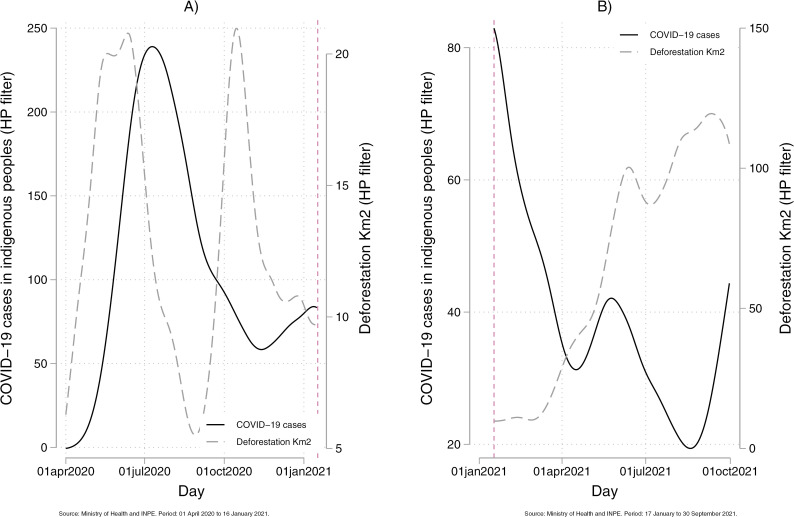
Correlation between confirmed COVID-19 cases in Indigenous peoples and deforestation: before and after vaccination. The solid line represents confirmed COVID-19 cases among Indigenous peoples from April 1, 2020, to January 16, 2021 (Fig 3A) and from January 17, 2021, to September 30, 2021 (Fig 3B). The dashed line represents the deforested area. Both datasets were smoothed using the Hodrick-Prescott (HP) filter, which removes short-term noise to highlight underlying trends. The vertical line marks the start of the vaccination program.

**Fig 3:**
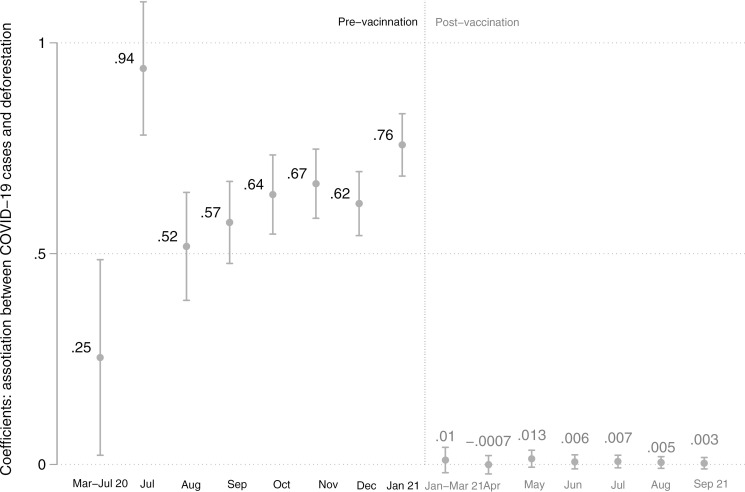
Estimated deforestation coefficients per month before and after vaccination. This graph presents the monthly estimated coefficients for the association between deforestation and COVID-19 based on the municipal-level sample and accumulated values. The estimations follow equation (1) but limit the time per month before and after vaccination. All independent variables are lagged by 14 days. Confidence intervals at the 95% level are represented as spikes, with p-values underlined: *** p < 0.01, ** p < 0.05, * p < 0.1.

**Fig 4:**
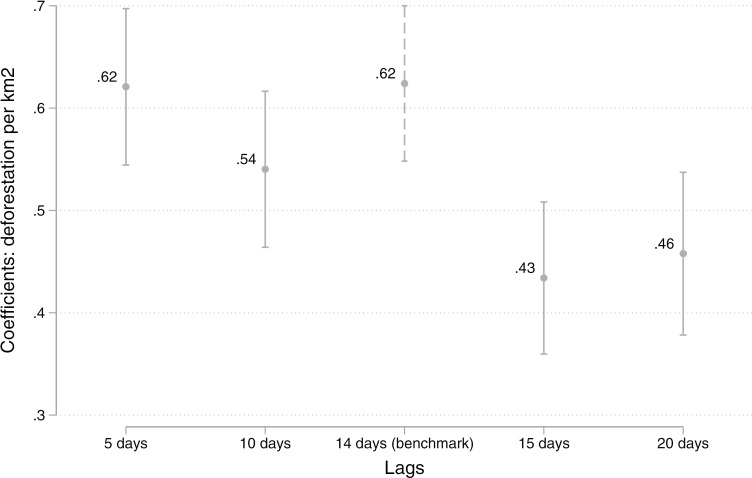
Sensitivity analysis: association between deforestation per km² and COVID-19 cases in Indigenous peoples using different lags.

The fixed-effects model, therefore, uses within-municipality or Indigenous health subdistrict variation over time for parameter identification. The model accounts for factors that do not change over time but vary across municipalities or Indigenous health subdistricts. By absorbing these unobserved characteristics, the fixed-effect model eliminates the bias when omitted variables correlate with the independent variable. The model also removes these unchanging factors, ensuring that such pre-existing differences do not bias estimates. Since fixed effects absorb all time-invariant factors, we do not need to include an extensive list of confounders. Another benefit of the current analysis is the possibility of adding lagged independent variables to the estimation to capture delayed effects [18].

In our estimation, the coefficient β represents the estimated association between deforestation and COVID-19 spread among Indigenous peoples, assuming that fixed effects and controls adequately account for other latent determinants of infection that are simultaneously correlated with deforestation.

One potential problem of fixed-effect analysis is spatial autocorrelation. If deforestation in one municipality is correlated with nearby areas’ deforestation, the assumption of independent and identically distributed (i.i.d.) residuals is violated. To circumvent this potential problem, we replicated our main estimation and corrected for spatial heteroskedasticity and autocorrelation (HAC) robust standard errors [19,20]. This model corrects for dependence in errors across space, dealing with potential spatial autocorrelation problems. More specifically, we reported results for spatial and serial correlation adjustment for regressions with high dimensional fixed effects [20].

### Mediation analysis

In addition to our panel data models, we estimate a cross-sectional model aggregating COVID-19 cases and deforestation over the study period. This allows us to examine potential mediation pathways through which deforestation contributes to Indigenous COVID-19 cases.

Previous studies suggest that deforestation is linked to conflicts between Indigenous and non-Indigenous peoples [23] and the expansion of illegal mining activities [24]. Additionally, deforestation has been associated with wildfires [25,26] and rainfall [27]. Socioeconomic factors, such as migration, appear to influence both deforestation [28] and COVID-19 transmission [29–31]. Furthermore, state capacity and public policy effectiveness are related to deforestation [32,33] and COVID-19 prevention [34]These interconnections suggest that deforestation may also contribute to an increase in COVID-19 infections through multiple economic, social, institutional, environmental, or health-related pathways.

To examine these potential pathways, we conducted a mediation analysis [21,22], using COVID-19 cases as the dependent variable and deforestation per km² as the independent variable. Mediation effects are tested by incorporating socioeconomic (illegal mining expansion, conflicts, GDP per capita, income inequality, Human Development Index, and expenditures during the COVID-19 pandemic), geographic (proximity to waterways, rainfall), and health system variables (average size of Indigenous health teams, number of hospitals) as potential mediators in the regression models. The mediation analysis was performed using cross-sectional data for the period preceding the rollout of the COVID-19 vaccination program. We first regress the mediator on the independent variable, then the outcome on both the independent variable and the mediator [22]. Finally, we estimate the Average Causal Mediation Effect (ACME) and Average Direct Effect (ADE). All estimations use the Ordinary Least Squares (OLS) model.

## Results

Fig 1 presents the spatial distribution of COVID-19 cases among Indigenous peoples and deforestation within Indigenous health subdistricts. Fig 2 illustrates the temporal trends in both variables. Between May 2020 and January 2021, COVID-19 cases among Indigenous peoples increased sharply, mirroring the rise in deforestation alerts. However, after the vaccination campaign began in January 2021, the relationship weakened—COVID-19 cases declined steadily, while deforestation continued to increase.

Table 2 reports the main regression results, estimating the association between deforestation and COVID-19 cases at the municipal and Indigenous health subdistrict levels. In the pre-vaccination period (Columns 1 & 2), we observe a positive and statistically significant association between deforestation and COVID-19 cases among Indigenous peoples. Before vaccination, a one-unit increase in daily deforestation per km² was associated with an average of 0.76 additional COVID-19 cases after 14 days (p < 0.004, 95% CI: 0.240 - 1.276, Table 1, Column 2). At the Indigenous health subdistrict level, a daily increase in deforestation per 100 km² was associated with an average of two additional COVID-19 cases after 14 days. The previously observed association disappears in the post-vaccination period (Columns 3 & 4), with coefficients no longer statistically significant. This suggests that vaccination mitigated the link between deforestation and COVID-19 spread among Indigenous populations.

**Table 2:** Fixed-effects results: deforestation and COVID-19 cases among Indigenous peoples before and after vaccination.

	Pre-vaccination		Post-vaccination		
	Dependent variable: COVID-19 in Indigenous peoples				
	Fixed- cases confirmed effects		Fixed-effects		
	Indigenous health subdistricts	Total municipalities	Indigenous health subdistricts	Total municipalities	
	(1)	(2)	(3)	(4)	
				
Deforestation per km^2^	0.02*	0.76***	0.00	0.00	
(lag 14 days)	0.015	0.264	0.008	0.012	
	[0.003]	[0.038]	[0.002]		[0.007]
	(-0.005 - 0.054)	(0.240 - 1.276)	(-0.015 - 0.015)		(-0.021 - 0.026)
	0.099	0.004	0.982		0.810
				
Implied cumulative effect	0.03	-0.76***	0.00		-0.01
(lag 14 days)	0.020	0.264	0.009		0.012
	[0.005]	[0.038]	[0.003]		[0.007]
	(-0.014 - 0.065)	(-1.278 - -0.242)	(-0.016 - 0.021)		(-0.030 - 0.016)
	0.200	0.004	0.815		0.547
				
Observations	101,371	1,499,566	89,386		65,752
R-squared	0.001	0.000	0.000		0.000

These findings indicate that while deforestation may have contributed to the initial spread of COVID-19 among Indigenous peoples, vaccination played a key role in breaking this association. The estimations incorporated robust spatial heteroskedasticity and autocorrelation (HAC) standard errors, suggesting that spatial dependence does not drive the estimated relationship between deforestation and COVID-19 transmission.

All columns present fixed-effects estimations incorporating time and municipality or Indigenous health subdistrict dummies with robust spatial heteroskedasticity and autocorrelation (HAC) standard errors. Columns 1 and 3 report results at the municipal level, while Columns 2 and 4 present results at the Indigenous health subdistrict level. The cumulative effect of deforestation is estimated as deforestation per 100 km²t ₋ ₁/ (1 - COVID-19 cases_t–1_). All independent variables are lagged by 14 days. HAC-robust standard errors are shown in the second row, followed by standard errors non-corrected by spatial autocorrelation in brackets in the third row. The fourth row shows the 95% confidence intervals. The p-values are in the last row of each variable, with significance levels indicated as follows: *** p < 0.01, ** p < 0.05, * p < 0.1.

We conducted monthly estimations from May 2020 to September 2021 to examine how the association between deforestation and COVID-19 cases among Indigenous peoples evolved before and after the vaccination program was implemented. Fig 3 illustrates that this association fluctuated over time, ranging from 0.26 (SE = 0.12) in June 2020 to 0.94 (SE = 0.08) in July 2020. However, from 17 January 2021 onward—the start of the vaccination campaign—the association lost statistical significance, suggesting that vaccination mitigated the impact of deforestation on COVID-19 transmission.

By the start of vaccination, deforestation alerts had accumulated to 3,979 km², and the total number of confirmed COVID-19 cases among Indigenous peoples reached 31,445. The average number of COVID-19 cases per km² at the municipal level was 7.90. Using the estimated coefficient from Table 1, Column 2, we calculate that approximately 9.6% of all confirmed COVID-19 cases among Indigenous peoples before vaccination can be attributed to deforestation.

### Sensitivity analysis and Robustness check

Using lagged variables for deforestation is a crucial component of our econometric specification, ensuring that we capture the time difference from the infection to the manifestation of symptoms. To test the robustness of our results, we conducted a sensitivity analysis by varying the deforestation lag structure from 5 to 20 days. Fig 4 demonstrates that the association between deforestation alerts and COVID-19 cases among Indigenous peoples remains statistically significant across different lag structures. However, the magnitude of the estimated coefficients decreases, on average, as the lag length increases, suggesting that the impact of deforestation on COVID-19 transmission is strongest within a shorter time window and gradually weakens over longer periods.

This figure presents the estimated coefficients (β) from Equation (1) using lag values ranging from 5 to 20 days. The benchmark lag is set at 14 days.

We also estimated the correlation between deforestation and COVID-19 cases for other racial groups in Brazil, including white, mixed (pardo), Asian, and black populations. Table 3 indicates that this association is statistically indistinguishable from zero for all non-Indigenous racial groups. This suggests that the observed relationship between deforestation and COVID-19 transmission is unique to Indigenous communities, potentially due to their higher exposure to deforestation-related risks, such as territorial displacement and increased interactions with external workers involved in deforestation-related activities.

**Table 3:** Fixed-effects results: testing for additional racial groups.

	Pre-vaccination			
	Dependent variable: COVID-19 cases confirmed			
	Fixed-effects			
	White	Mixed	Asian	Black
	(1)	(2)	(3)	(4)
Deforestation per km^2^	0.01	0.00	-0.00	-0.00
(lag 14 days)	0.040	0.025	0.001	0.004
	(-0.066 - 0.092)	(-0.044 - 0.052)	(-0.003 - 0.003)	(-0.011 - 0.005)
	0.751	0.869	0.909	0.460
				
Implied cumulative effect	-0.02	-0.01	-0.00	-0.00
(lag 14 days)	0.032	0.024	0.001	0.004
	(-0.086 - 0.038)	(-0.060 - 0.033)	(-0.003 - 0.003)	(-0.012 - 0.003)
	0.445	0.579	0.899	0.253
Observations	565,746	573,828	759,835	724,426
R-squared	0.000	0.000	0.000	0.000
Number of municipalities	2,879	2,879	2,879	2,879

This table presents fixed-effects estimations assessing the association between deforestation and COVID-19 cases among additional racial groups—White (1), Mixed (2), Asian (3), and Black (4). The results indicate no significant association. All models include time and municipality fixed-effects at the municipal level.

### Mediation analysis

**Fig 5:**
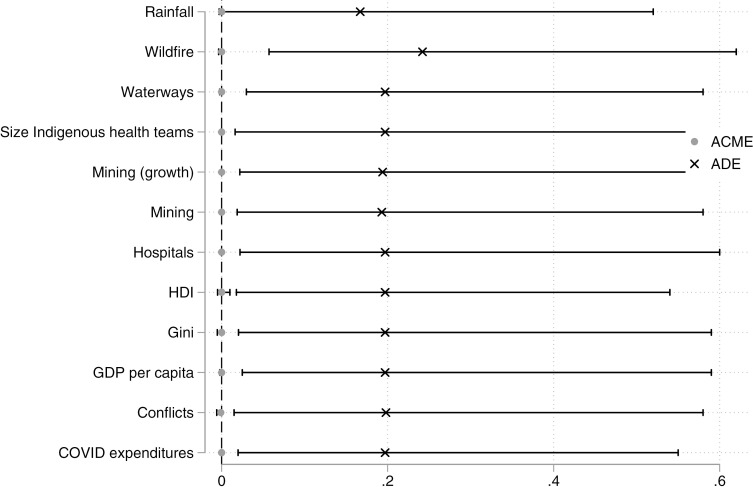
Mediation analysis. This graph presents the results of a mediation analysis examining 12 potential mediators in the relationship between deforestation and COVID-19 infections among Indigenous peoples. The mediators include wildfire, distance to waterways, Indigenous health team size, growth in illegal mining activities, presence of illegal mining, Human Development Index, number of hospitals, income inequality (Gini Index), GDP per capita, conflicts between Indigenous and non-Indigenous peoples, and COVID-19 expenditures during the pandemic. Each mediator has two estimated effects: Average Causal Mediation Effects (ACME) and Average Direct Effects (ADE). The ACME represents the mediator’s indirect effect, indicating whether mediation is statistically significant.

The results in Fig 5 indicate that none of the examined variables functioned as statistically significant mediators of COVID-19 transmission through deforestation in our cross-sectional analysis. The Average Causal Mediation Effects (ACME) reported are all indistinguishable from zero. Only the direct effect (ADE) demonstrates explanatory power.

### Vaccination

**Fig 6:**
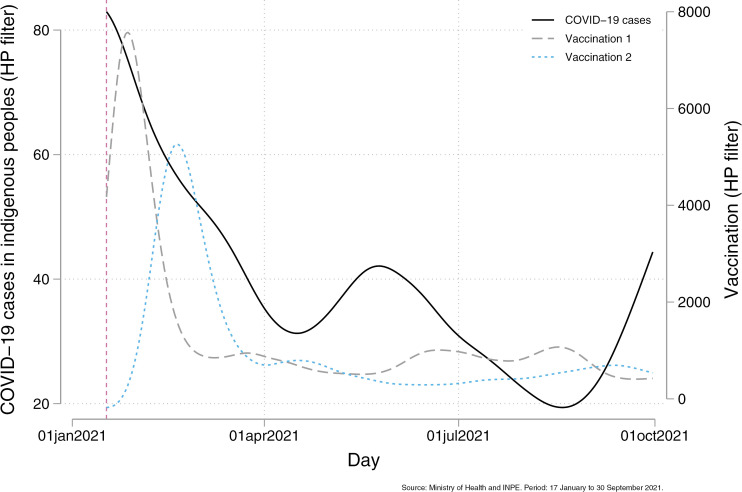
COVID-19 cases and vaccination among Indigenous peoples. This figure presents the relationship between confirmed COVID-19 cases and vaccination among Indigenous peoples from January 17, 2021, to September 30, 2021. The solid line represents confirmed COVID-19 cases. The gray dashed line indicates the first dose of the COVID-19 vaccine administered, while the blue dashed line represents the second dose. All three series were smoothed using the Hodrick-Prescott (HP) filter, which removes short-term noise to reveal underlying trends. The vertical dashed line marks the start of the vaccination program.

The COVID-19 vaccination campaign for Indigenous peoples in Brazil began on 16 January 2021. From this point forward, the previously observed association between deforestation and COVID-19 transmission among Indigenous communities disappeared. Fig 6 illustrates the inverse correlation between vaccination uptake and COVID-19 cases, showing a steady decline in infection as vaccination coverage increased.

These findings highlight the critical role of vaccination in mitigating the indirect health risks associated with environmental degradation and deforestation, reinforcing the importance of targeted public health interventions for vulnerable populations.

## Discussion

Our statistical analysis demonstrated that deforestation was significantly associated with the transmission of COVID-19 among Brazil’s Indigenous peoples in the absence of vaccination. This finding underscores an additional negative externality of deforestation during the pandemic, disproportionately affecting one of the most vulnerable segments of society. Our results also provide empirical evidence of a One Health case, in which environmental degradation directly contributes to the spread of a pandemic infectious disease. This dynamic adds complexity to the preparedness and response capacity of the already fragmented and under-resourced Indigenous health system, highlighting the urgent need for integrated health and environmental policies.

Despite the robustness of our findings, our study has limitations. First, while we consistently estimated the association between COVID-19 transmission and deforestation, our municipal-level analysis benefits from a larger sample size and richer covariates. Still, it assumes that COVID-19 affected municipalities within Indigenous Special Sanitary Districts (ISSDs) in proportion to their population size. To address this, we also conducted an Indigenous health subdistrict-level analysis, where data is directly reported and not subject to redistribution assumptions.

Second, even after employing several robustness checks, we were unable to identify the exact mechanisms driving COVID-19 transmission following deforestation. A key challenge is that it is difficult to observe social interactions that may have led to increased contact between infected and non-infected individuals due to deforestation. Some authors have used cell phone data to observe mobility [35], but this is not a feasible estimation strategy for this study because the data does not distinguish racial categories. Forced displacement resulting from increased deforestation and wildfires has been highlighted by Indigenous leaders and organizations as a major concern since the pandemic’s inception [26]. However, available data did not empirically confirm a mediation effect. These findings were unexpected. Prior research suggests that territorial conflicts, illegal mining, and forced migration have intensified since 2019, coinciding with increased deforestation and the exploitation of natural resources in Indigenous lands. However, our quantitative analysis remains inconclusive regarding the precise mechanisms by which deforestation may have contributed to increased COVID-19 transmission among Indigenous peoples. Further research is needed to explore alternative transmission pathways.

Lastly, our results must be interpreted within the broader epidemiological, institutional, political, geographical, environmental, and cultural contexts. While the findings are significant for Brazil, external validity requires further investigation in other Amazonian countries, such as Peru and Colombia, where deforestation, Indigenous vulnerability, and health inequities may have played similar roles in disease transmission. Future research should replicate this analysis across different socio-ecological settings to strengthen comparative insights.

Our results highlight the critical need for public policies that integrate health and environmental dimensions to effectively combat disease transmission. The inclusion of natural systems in public health strategies is at the core of One Health, Planetary Health, and Indigenous Health approaches [16,27]. These frameworks recognize that human health is intrinsically linked to ecosystem health, and policies must reflect this interconnectedness.

The One Health approach, endorsed by global health organizations, advocates for cross-sectoral collaboration between public health, environmental science, and veterinary medicine to prevent zoonotic spillovers and mitigate future pandemics. Our findings reinforce that deforestation and land-use change create conditions conducive to disease emergence and spread, necessitating stricter environmental governance as a public health measure.

At the global level, deforestation regulation is increasingly recognized as a critical component of pandemic prevention. Initiatives such as the EU Deforestation-Free Regulation (EUDR) and the Glasgow Leaders’ Declaration on Forests and Land Use seek to curb deforestation-driven biodiversity loss and emerging health threats. However, these measures not binding and do not explicitly integrate public health goals.

In the Brazilian context, deforestation policies must align with Indigenous rights and health protections. The dismantling of environmental safeguards during the pandemic exacerbated vulnerabilities, leading to increased land invasions, illegal mining, and displacement. Strengthening environmental monitoring, Indigenous land protections, and enforcement of anti-deforestation laws is crucial for biodiversity conservation and preventing health crises.

The strong association between deforestation and COVID-19 transmission among Indigenous peoples before vaccination underscores the necessity of broadening the scope of health policy to incorporate environmental and social justice dimensions. The pandemic has exacerbated existing inequities, disproportionately impacting marginalized groups. A sustainable and equitable recovery must prioritize integrated health, environmental, and Indigenous rights-based policies to enhance resilience against future health and ecological crises.

## Supporting information

S1 File Description and source of the variables S1.2. Dependent variables. S1.3. Main independent variables. S1.4. Control variables used in the cross-section analysis. S1.5. Spatial data.
(DOCX)

Fig S1 Estimated Deforestation Coefficients Per Month Before and After Vaccination. (EPS)

Fig S2 Estimated Deforestation Coefficients Per Month Before and After Vaccination using 6 lags. (EPS)
