# Supplementary material for: The effect of deforestation on COVID-19 transmission to Indigenous peoples in Brazil: A panel fixed-effects analysis before and after vaccination
Source: PLOS Glob Public Health. 2025 Apr 29;5(4):e0004527. doi: 10.1371/journal.pgph.0004527 (PMC12040269; doi:10.1371/journal.pgph.0004527)
Supplement: S1 File — Dependent variables. S1.3. Main independent variables. S1.4. Control variables used in the cross-section analysis. S1.5. Spatial data. (DOCX) [file pgph.0004527.s001.docx]

**The effect of deforestation on COVID-19 transmission to Indigenous peoples in Brazil: a panel fixed-effects analysis before and after vaccination**

Humberto Laudares, PhD, Carolina Batista, MD, Pedro Henrique Gagliardi, MPA, Prof. Rudi Rocha, PhD and Prof. Nicolas Ray, PhD

**Supplementary Material**

# Supplementary text S1. Description and source of the variables

S1.2. Dependent variables

- Daily number of COVID-19 cases for Indigenous peoples reported by the Special Secretariat for Indigenous Health (SSIH) within the Brazilian Unified Health System (SUS) from 1 April 2020 to 30 September 2021.
  - The database obtained counted the number of infections throughout the country's 34 Special Indigenous Health Districts (ISSD) and its Indigenous health subdistricts (*pólo base*).
  - We run the analysis at Indigenous health subdistricts and at the municipal level.
  - For municipal-level analysis, we proportionally distributed the number of cases for each municipality located at least in one of these ISSDs (The distribution among Special Indigenous Health Districts (ISSDs) and municipalities).
  - We applied a relative frequency based on the estimates for the Indigenous population in each of these municipalities to find the corresponding proportion and, thus, determine the number of COVID-19 cases from the ISSDs at the municipal level, as follows:

$$\mathrm{COVIDcases}\left( x,y \right)=y_{i,d}\frac{x_{i}}{\sum_{n=j} x_{j}}$$

- - Where (y) is the daily number (d) of infected people in a certain ISSD that contains a determined municipality (i); (x) is the indigenous population of this municipality; and (j) is the set of all municipalities that are found in this specific ISSD.
  - Link: https://infoms.saude.gov.br/extensions/sesai_covid_v2/sesai_covid_v2.html
  - Access date: 18 January 2025.

S1.3. Main independent variables

- Deforestation: The Real-Time System for Detection of Deforestation (DETER) uses remote sensing technology to generate satellite imagery of forest cover. The system identifies deforestation hot spots and issues georeferenced deforestation alerts. We use the daily number of warning areas for deforestation (per km^2^) within the Amazon and the Cerrado biomes at the municipal level from 1 April 2020 to 30 September 2021. We selected the data that only referred to deforestation, excluding other alerts, such as mining. We also use the data from Indigenous health subdistricts based on the author's own calculation using GIS.
  - Data collected from the Deter database produced by Brazil’s National Institute for Space Research.
  - Link: <http://terrabrasilis.dpi.inpe.br/en/home-page/>
  - Access date: 17 December 2023.
- Vaccination: Daily number of indigenous people vaccinated against COVID-19 (1^st^ and 2^nd^ doses) per municipality from 17 January to 30 September 2021.
  - Data extracted from the Ministry of Health’s database of the National Vaccination Campaign against COVID-19.
  - Link: <https://opendatasus.saude.gov.br/dataset/covid-19-vacinacao>
  - Access date: 15 September 2023.
- Rainfall: Daily precipitation volume per municipality from 17 January to 30 September 2021.
  - Reference: Xavier, A. C., Scanlon, B. R., King, C. W., & Alves, A. I. (2022). New improved Brazilian daily weather gridded data (1961–2020). International Journal of Climatology, 42(16), 8390–8404.
  - Link: https://sites.google.com/site/alexandrecandidoxavierufes/brazilian-daily-weather-gridded-data
  - Access date: 25 September 2023.
- Wind speed: Daily average wind speed per municipality from 17 January to 30 September 2021.
  - Reference: Xavier, A. C., Scanlon, B. R., King, C. W., & Alves, A. I. (2022). New improved Brazilian daily weather gridded data (1961–2020). International Journal of Climatology, 42(16), 8390–8404.
  - Link: https://sites.google.com/site/alexandrecandidoxavierufes/brazilian-daily-weather-gridded-data
  - Access date: 25 September 2023.

S1.4. Control variables used in the cross-section analysis

- Geographical variables: produced with the ArcGIS software based on available public shapefiles.
  - Rainfall: Average rainfall at the municipal level in millimeters per hour (mm).
  - Waterway: Binary variable for municipalities whose centroids are at least 100 km from the nearest waterway.
  - Link: <https://www.ibge.gov.br/geociencias/downloads-geociencias.html>
  - Access date: 13 September 2020.
- Illegal mining
  - Binary variable for municipalities that exhibit illegal mining activities. Data was updated in 2020.
    - The Amazon Geo-Referenced Socio-Environmental Information Network (RAISG) compiled the data.
    - Links: https://mineria.amazoniasocioambiental.org/sobre/.
    - Access date: 08 September 2021
  - The number of km^2^ of illegal mining area opened between 2019 and 2020.
    - The data produced by MapBiomas. The data are public, open, and free under the Creative Commons Attribution-ShareAlike (CC BY-SA) license.
    - Link: https://mapbiomas.org/download.
    - Access date: 30 November 2021.
- Conflicts.
  - Number of land conflicts: Total number of land conflicts involving Indigenous people in Brazil in 2019.
    - Data collected by the Commissão Pastoral da Terra.
    - Link: https://www.cptnacional.org.br/.
    - Access date: 27 November 2020.
- Cattle ranching.
  - The total number of bovine cattle by municipality per 1,000 km^2^ in 2019. We also used it in natural logarithmic form.
    - Data from the Brazilian Institute of Geography and Statistics (IBGE).
    - Link:https://www.ibge.gov.br/estatisticas/economicas/agricultura-e-pecuaria/21814-2017-censo-agropecuario.html?=&t=o-que-e.
    - Access date: 13 September 2020.
- Wildfire
  - Wildfire is captured by the Fire Radiative Power (FRP), which is the measurement of the radiant energy released per time unit by burning vegetation per 1,000 km^2^ from 1 April 2000 to 31 August 2021. We also used the data in natural logarithmic form.
    - The data from the National Institute for Space Research Burning Program.
    - Link: <http://queimadas.dgi.inpe.br/queimadas/portal>.
    - Access date: 30 November 2024.
- Health systems variables
  - Average size of Indigenous health teams.
  - Number of hospitals.
    - The data is from the Ministry of Health DataSUS.
    - Link: https://datasus.saude.gov.br/
    - Access date: 13 October 2021.
- Economic Variables
  - Gross Domestic Production (GDP) per capita (2017).
  - Income inequality (Gini Index) (2010).
    - The data is from the Brazilian Institute of Geography and Statistics (IBGE).
    - Link: www.ibge.gov.br
    - Access date: 17 August 2023.
  - Human Development Index (HDI)
    - The HDI is the geometric mean of normalized indices that captures three dimensions: a long and healthy life, being knowledgeable, and having a decent standard of living. The data from 2010.
    - The United Nations Development Programme (UNDP) produced the data, but it was retrieved from IPEAdata.
    - Link: [www.ipeadata.gov.br](http://www.ipeadata.gov.br)
    - Access date: 02 May 2020.
  - Expenditure related to COVID-19
    - The total amount transferred from the central government to the local government during the COVID-19 pandemic in 2020.
    - The data is produced by the Treasury Department from the Ministry of Finance.
    - Link: https://www.tesourotransparente.gov.br/
    - Access date: 13 October 2021.

S1.5. Spatial Data

The spatial data plotted in Fig 1 were obtained from freely accessible and reusable sources of the Brazilian government. Further details are listed below:

- Administrative borders
  - Brazilian and state borders shapefiles were retrieved from IBGE (Instituto Brasileiro de Geografia e Estatística), and they are under the Creative Commons Attribution-ShareAlike 4.0 International (CC BY-SA 4.0) license.
  - The Indigenous health subdistricts shapefile was requested to the Ministry of Health, Department of Indigenous Health, through the LAI—Law on Access to Information, No. 12.527/2011. We received the shapefile under protocol number 25072.003130/2024-67 on 02/02/2024. The protocol stated that the information is not restricted and, therefore, is in the public domain.
- Deforestation
  - Data collected from the Deter database produced by Brazil’s National Institute for Space Research.
  - Link: http://terrabrasilis.dpi.inpe.br/en/home-page/
  - Access date: 17 December 2023.
  - This INPE dataset is licensed under Creative Commons Attribution-ShareAlike 4.0 International (CC BY-SA 4.0).
- COVID-19 daily cases in Indigenous peoples
  - The database obtained counted the number of infections throughout the country's 34 Special Indigenous Health Districts (ISSD) and its Indigenous health subdistricts (*pólo base*).
  - Link: https://infoms.saude.gov.br/extensions/sesai_covid_v2/sesai_covid_v2.html
  - Access date: 18 January 2025.
  - The Brazilian Ministry of Health data is in the public domain, allowing free use and distribution.

**Fig S1**. **Estimated Deforestation Coefficients Per Month Before and After Vaccination.** This graph presents the monthly estimated coefficients for the association between deforestation and COVID-19 based on the Indigenous health subdistrict sample and accumulated values. The estimations follow equation (1) but limit the time per month before and after vaccination. All independent variables are lagged by 14 days. Confidence intervals at the 95% level are represented as spikes, with p-values underlined: *** p<0.01, ** p<0.05, * p<0.1.

**Fig S2**. **Estimated Deforestation Coefficients Per Month Before and After Vaccination using 6 lags.** This graph presents the monthly estimated coefficients for the association between deforestation and COVID-19 based on the municipal-level sample and accumulated values. The estimations follow equation (1) but limit the time per month before and after vaccination. All independent variables are lagged by 6 days. Confidence intervals at the 95% level are represented as spikes, with p-values underlined: *** p<0.01, ** p<0.05, * p<0.1.

# Table S1. Summary statistics: panel data at municipal level

|  |  |  |  |  |  |  |
| --- | --- | --- | --- | --- | --- | --- |
| **Variables** | **Number of variables** | **Mean** | **Standard error** | **Minimum** | **Maximum** | **Sum** |
|  |  |  |  |  |  |  |
| COVID-19 cases | 1,802,052 | 0.023 | 0.401 | 0 | 72 | 41,349 |
| Deforestation (in Km2) | 1,802,052 | 0.012 | 0.381 | 0 | 119 | 21,958 |
| Vaccination (1st dose) | 1,802,052 | 0.200 | 4.108 | 0 | 1,126 | 359,614 |
| Vaccination (2nd dose) | 1,802,052 | 0.142 | 3.177 | 0 | 758 | 256,575 |
| Rainfall (daily) | 1,802,052 | 10.547 | 58.343 | 0 | 1,305 | 19,000,000 |
| Wind speed (daily) | 1,801,306 | 1.997 | 1.192 | 0.002 | 12 | 3,597,317 |
|  |  |  |  |  |  |  |

Notes: The table shows the main variables used for the panel data analysis, namely COVID-19 cases confirmed in Brazil’s indigenous peoples, deforestation alerts in km^2^, the number of 1^st^ and 2^nd^ doses administered, the daily precipitation volume, and wind speed.

# Table S2. Summary statistics: panel data at Indigenous health subdistricts

|  |  |  |  |  |  |  |
| --- | --- | --- | --- | --- | --- | --- |
| **Variables** | **Number of variables** | **Mean** | **Standard error** | **Minimum** | **Maximum** | **Sum** |
|  |  |  |  |  |  |  |
| COVID-19 cases | 209,598 | 0.197 | 1.062 | 0 | 37 | 41,349 |
| Deforestation (in Km2) | 209,598 | 0.102 | 2.483 | 0 | 341 | 21,958 |
|  |  |  |  |  |  |  |
|  |  |  |  |  |  |  |

Notes: The table shows the main variables used for the panel data analysis, namely COVID-19 cases confirmed in Brazil’s Indigenous peoples and deforestation alerts in km2 at the Indigenous health subdistrict level.

# Table S3. Summary statistics for cross-section data

|  |  |  |  |  |  |  |
| --- | --- | --- | --- | --- | --- | --- |
| **Variables** | **Number of variables** | **Mean** | **Standard error** | **Minimum** | **Maximum** | **Sum** |
|  |  |  |  |  |  |  |
| COVID cases | 5,570 | 7.42 | 54.62 | 0.00 | 1,975.00 | 41,349.00 |
| COVID-19 cases until 16 January 2021 | 5,570 | 5.65 | 46.68 | 0.00 | 1,661.00 | 31,445.00 |
| COVID-19 cases from 17 January until 30 September 2021 | 5,570 | 1.78 | 15.40 | 0.00 | 439.00 | 9,904.00 |
| Deforestation per Km2 | 5,570 | 3.94 | 26.78 | 0.00 | 753.25 | 21,957.91 |
| Deforestation until 16 January 2021 | 5,570 | 0.71 | 4.98 | 0.00 | 173.24 | 3,979.08 |
| Deforestation from 17 January until 30 September 2021 | 5,570 | 3.23 | 26.28 | 0.00 | 749.81 | 17,978.83 |
| Existence of conflicts between indigenous and non-indgenous (CPT) | 5,385 | 0.04 | 0.19 | 0.00 | 1.00 | 199.00 |
| Area illegal mining (2019-2020) | 5,570 | 2.11 | 57.36 | -37.93 | 3,694 | 11,771 |
| Growth in illegal mining during 2019-2020 | 5,570 | 1.87 | 75.95 | -65.50 | 5,433 | 10,402 |
| Wildfires | 4,381 | 3.29 | 0.71 | 0.88 | 6.52 | 14,414 |
| Cattle ranching | 5,332 | 9.30 | 1.48 | 3.04 | 14.27 | 49,608 |
| Rainfall | 5,039 | 11.64 | 5.33 | 0.00 | 33.00 | 58,638 |
| Waterways | 5,383 | 0.05 | 0.23 | 0.00 | 1.00 | 294 |
| Latitude | 5,383 | -16.50 | 8.31 | -33.69 | 4.60 | -88,804 |
| Size of Indigenous health teams | 5,385 | 0.05 | 0.46 | 0.00 | 18.00 | 264 |
| Number of hospitals | 5,382 | 0.97 | 3.27 | 0.00 | 150 | 5,205 |
| GDP per capita (2017) | 5,383 | 22,142 | 21,068 | 3,285 | 344,847 | 119,000,000 |
| Income inequality (Gini coefficient) | 5,378 | 0.50 | 0.07 | 0.28 | 0.81 | 2,705.38 |
| Human Development Indicator (HDI) | 5,378 | 0.66 | 0.07 | 0.42 | 0.86 | 3,550.58 |
| Expenditure related to COVID-19 | 5,385 | 698,855 | 3,099,437 | 0.00 | 89,800,000 | 3,760,000,000 |
|  |  |  |  |  |  |  |

Notes: The table shows the main variables used for the cross-section analysis.

# Table S4. Fixed-effects results: controlling for climate and clustering standard errors

|  |  |  |  |  |  |  |  |
| --- | --- | --- | --- | --- | --- | --- | --- |
|  | **Pre-vaccination** | | |  | **Post-vaccination** | | |
|  | Dependent variable: COVID-19 cases confirmed in Indigenous Peoples | | | | |  |  |
|  | Fixed-effects | | |  | Fixed-effects | | |
|  | Indigenous health subdistricts |  | Total municipalities |  | Indigenous health subdistricts |  | Total municipalities |
|  | (1) |  | (2) |  | (3) |  | (4) |
|  |  |  |  |  |  |  |  |
| Deforestation per Km2 | 0.02 |  | 0.76*** |  | 0.00 |  | 0.00 |
| (lag 14 days) | 0.02 |  | 0.173 |  | 0.005 |  | 0.013 |
|  | (-0.014 - 0.064) |  | (0.419 - 1.096) |  | (-0.009 - 0.010) |  | (-0.023 - 0.029) |
|  | 0.210 |  | 0.000 |  | 0.972 |  | 0.831 |
|  |  |  |  |  |  |  |  |
| Implied cumulative effect | 0.03 |  | -0.76*** |  | 0 |  | -0.01 |
| (lag 14 days) | 0.019 |  | 0.173 |  | 0.007 |  | 0.012 |
|  | (-0.011 - 0.063) |  | (-1.099 - -0.421) |  | (-0.011 - 0.016) |  | (-0.031 - 0.017) |
|  | 0.172 |  | 0.000 |  | 0.749 |  | 0.565 |
|  |  |  |  |  |  |  |  |
| Observations | 101,371 |  | 1,498,919 |  | 89,386 |  | 65,751 |
| R-squared | 0.001 |  | 0.003 |  | 0.000 |  | 0.016 |
| Clustered standard errors | Yes |  | Yes |  | Yes |  | Yes |
| Climate controls | No |  | Yes |  | No |  | Yes |
| Number of Indigenous health subdistricts | 362 |  |  |  | 362 |  |  |
| Number of municipalities |  |  | 5,569 |  |  |  | 2,049 |
|  |  |  |  |  |  |  |  |

# Notes: All columns present fixed-effects estimations, controlling for time and municipality dummies at municipal and Indigenous health subdistrict levels. Columns 1 and 3 report results at the Indigenous health subdistrict level, while Columns 2 and 4 present results at the municipal level. Columns 1 and 3 have standard errors clustered by the Indigenous health subdistrict. Columns 2 and 4 control for climate variables -- rainfall and wind speed – and have standard errors clustered by municipality. The cumulative effect of deforestation is estimated as deforestation per 100 km²t₋₁ / (1 - COVID-19 casesₜ₋₁). All independent variables are lagged by 14 days. Standard errors are shown in the second row, followed by the 95% confidence intervals in the third row. In the fourth row, p-values are underlined, with significance levels indicated as follows: *** p<0.01, ** p<0.05, * p<0.1.
